# Molecular Characterization of Hemoparasites and Hemoplasmas Infecting Domestic Cats of Southern India

**DOI:** 10.3389/fvets.2020.597598

**Published:** 2021-01-25

**Authors:** Lanchalung Malangmei, Karapparambu Gopalan Ajith Kumar, Ashwathappa Nandini, Christophe Angeline Felicia Bora, Anju Varghese, Birur Mallappa Amrutha, Prashant Somalingappa Kurbet, Rangapura Kariyappa Pradeep, Murikoli Nimisha, Chundiyil Kalarickal Deepa, Lijo John, Reghu Ravindran

**Affiliations:** ^1^Department of Veterinary Parasitology, College of Veterinary and Animal Sciences, Wayanad, India; ^2^Department of Veterinary Biochemistry, College of Veterinary and Animal Sciences, Wayanad, India

**Keywords:** cats, *Cytauxzoon* spp., *Hepatozoon felis*, *Candidatus* M. haemominutum, Mycoplasma haemofelis, *Candidatus* M. turicensis, phylogeny

## Abstract

In the present study, 111 blood samples were collected from apparently healthy cats belonging to four districts of Kerala, southern India, and they were investigated for the presence of hemoparasites and hemoplasmas by light microscopic examination and polymerase chain reaction (PCR). The microscopic examination of the Giemsa-stained blood smears did not reveal any parasites/organisms. However, PCR followed by nucleotide sequencing could detect 10 (9.01%) out of 111 samples infected with *Hepatozoon felis*, 3 (2.70%) with *Cytauxzoon* spp., and 10 (9.01%) with *Mycoplasma* spp. None of the samples revealed amplicons specific for the *Babesia* spp. and *Trypanosoma evansi*. The phylogenetic analysis of 18S ribosomal RNA (rRNA) gene sequences of *H. felis* revealed the existence of two different populations of *H. felis* circulating in the blood of infected cats. The phylogenetic tree was constructed based on 18S rRNA gene sequences of *Cytauxzoon* spp. and revealed that these isolates formed a distinct clade and do not cluster with any of the isolates from other countries. Among the 10 samples positive for *Mycoplasma* spp. infections, 7 were detected positive for *Candidatus* Mycoplasma haemominutum, two for *Mycoplasma haemofelis*, and one for *Candidatus* Mycoplasma turicensis. Phylogenetic analysis of 16S rRNA gene sequences of *Mycoplasma* spp. showed no distinct geographical grouping of the sequences. The sequences of *M. haemofelis, Candidatus* M. haemominutum, and *Candidatus* M. turicensis identified in the study clustered along with their respective isolates from around the world. To the best of our knowledge, this study forms the first report of molecular detection of *Cytauxzoon* spp. and *Candidatus* M. turicensis in cats from India.

## Introduction

Diseases caused by hemoprotozoan and hemoplasmal organisms are emerging problems in cats in many parts of the world (1, 2). These diseases are mostly fatal vector-borne diseases and are quickly disseminated (1, 3). The increased occurrence of feline vector-borne diseases in Europe was speculated for the reasons like climatic change, increased vector population, drug resistance in vector/pathogen population, and increased international transport of man and animals (3, 4).

Feline hemoprotozoans include hemogregarines like *Hepatozoon felis, Hepatozoon canis*; piroplasm-causing organisms such as *Cytauxzoon felis, Cytauxzoon manul, Babesia felis, Babesia cati, Babesia herpailuri*, and *Babesia vogeli*; and hemoflagellates such as *Trypanosoma evansi, Trypanosoma cruzi, Trypanosoma brucei, Trypanosoma congolense*, and *Trypanosoma rangeli* (5, 6). *Mycoplasma haemofelis, Candidatus* Mycoplasma haemominutum, *Candidatus* Mycoplasma turicensis, and *Candidatus* Mycoplasma haematoparvum-like (1) are the hemotropic mycoplasmas. Among them, *M. haemofelis* and *Candidatus* M. haemominutum were originally classified under Anaplasmataceae, order Rickettsiales, and were previously known as *Haemobartonella felis* large and small forms, respectively (7, 8).

Cats having access to the outdoors are more vulnerable to these infections as a result of exposure to a variety of ectoparasites that may transmit these diseases (1, 9, 10). Ticks play an important role in the transmission of babesiosis, hepatozoonosis, and cytauxzoonosis (11–13). Bloodsucking insects such as *Tabanus, Stomoxys, Atylotus*, and *Lyperosia* act as vectors for trypanosomosis (14). Feline hemoplasmas are transmitted by *Ctenocephalides felis*, blood transfusion, and also by fighting, scratching, and biting with other cats. Other routes of infection include transuterine and transmammary transmission (1).

The population of pet cats in India was estimated to be around 2 million in 2018 (https://www.statista.com/statistics/1061172/india-population-of-pet-cats). The majority of them are feral or semidomesticated, which wander in the area around the house from where they may get food and shelter. Rearing domestic cats with a good pedigree has increased recently.

There were only a few documented reports on the molecular detection of these infectious agents in cats from India (15, 16). Hence, the present communication focuses on the generation of baseline information regarding the presence and distribution of hemoparasites and hemoplasmas in cats of southern India.

## Materials and Methods

### Study Area and Samples

**Figure 1:**
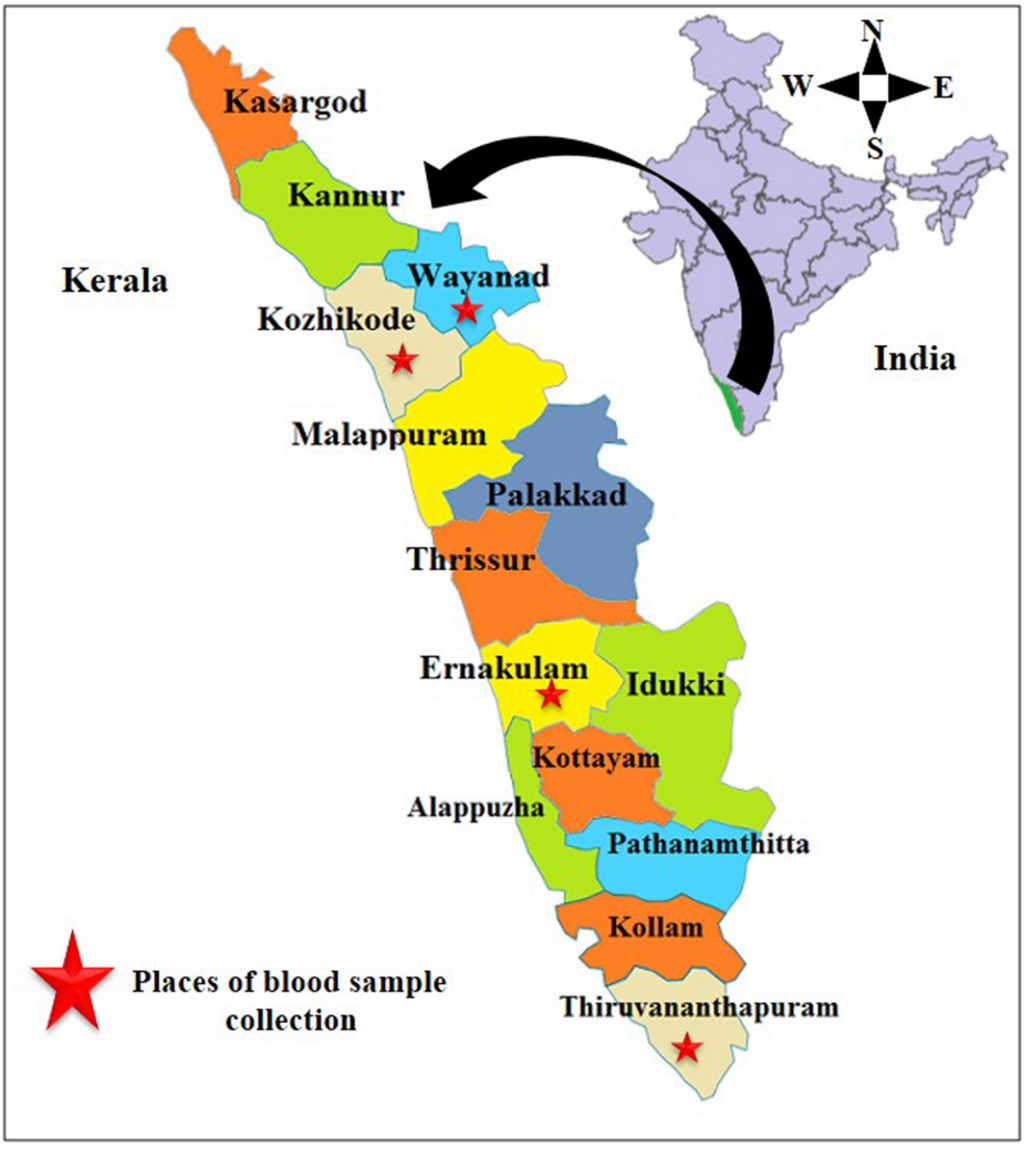
Places of collection of blood samples.

For this study, 111 blood samples were collected from cats belonging to four districts of Kerala, India, *viz*., Wayanad, Kozhikode, Ernakulam, and Thiruvananthapuram (Figure 1) in the period between March 2018 and June 2019. Whole blood samples were collected in ethylenediaminetetraacetic acid (EDTA) vials from femoral or medial saphenous veins. Thin blood smears were prepared using a drop of this blood.

### Staining

Thin peripheral blood smears were fixed in methanol, then stained with diluted (1:10) Giemsa's stain (Merck Life Science, Mumbai) for 45 min. The blood smears were washed with water and air dried. The stained blood smears were examined under the oil immersion objective (100× ) of the light microscope (Leica DM1000 LED, Germany) for the presence of parasites. A minimum of 150 fields were examined thoroughly before declaring a sample as negative.

### Genomic DNA Extraction and Quantification

Genomic DNA was isolated from blood samples collected in EDTA vials using DNeasy® blood and tissue kit (Qiagen, Germany), according to the manufacturer's protocol. Extracted DNA was eluted in 100 μL of DNA elution buffer. The DNA concentration was determined using a NanoDrop® 2000C spectrophotometer (Thermo Scientific, USA) and stored at −20°C for further analysis.

### Polymerase Chain Reaction

The genomic DNAs isolated from these samples were used for PCR, in an automated thermal cycler with a heated lid (Eppendorf, Germany). All PCRs were carried out in a final reaction volume of 25 μL containing 0.2 mM deoxyribonucleotide triphosphates (dNTPs) (Thermoscientific, Lithuania), 1 U DyNAzyme II DNA polymerase (Thermo Scientific, USA), 10×PCR buffer (containing MgCl_2_ at a final concentration of 1.5 mM), 20 ng of template DNA, and 10 pmol each of forward and reverse primers. The details of the primers used, the amplification conditions, and the amplicon size are shown in Table 1.

**Table 1:** The details of primers used for the PCR, the amplification conditions, and the amplicon size.

**Sl No**.	**Organism**	**Primer name**	**Primer sequence**	**Target**	**Amplification conditions**	**Amplicon size (bp)**	**Reference**
1	*Hepatozoon* spp. and *Cytauxzoon* spp.	Piroplasmid-F Piroplasmid-R	Forward, 5′CCAGCAGCCGCGGTAATT 3′ Reverse, 5′CTTTCGCAGTAGTTYGTCTTTAACAAATCT 3′	18S rRNA gene	Initial denaturation: 94°C for 3 min 35 cycles of 94°C for 30 s 64°C for 45 s 72°C for 30 s Final extension: 72°C for 7 min	358 bp	(17)
2	*Babesia* spp.	P_18S1F	Forward, 5′AAGATTAAGCCATGCATGTCTAA 3′	18S rRNA gene	Initial denaturation: 95°C for 5 min 60 cycles of 94°C for 1 min 50°C for 1 min 72°C for 1 min Final extension: 72°C for 10 min	1,612 bp	(18)
		P_18S1612R	Reverse, 5′AGTGATAAGGTTCACAAAACT T 3′				
3	*Hepatozoon* spp.	18SHepF	Forward, 5′ATACATGAGCAAAATCTCAAC3′	18S rRNA gene	Initial denaturation: 95°C for 5 min 34 cycles of 95°C for 30 s 57°C for 30 s 72°C for 90 s Final extension: 72°C for 5 min	~666 bp	(19)
		18SHepR	Reverse, 5′CTTATTATTCCATGCTGCAG3′				
4	*Cytauxzoon felis*	Cytz F	Forward, 5′GCGAATCGCATTGCTTTATGCT 3′	18S rRNA gene	Initial denaturation: 95°C for 5 min 40 cycles of 95°C for 45 s 59°C for 45 s 72°C for 60 s Final extension: 72°C for 5 min	284 bp	(20)
		Cytz R	Reverse, 5′ CCAAATGATACTCCGGAAAGAG 3′				
5	*T. evansi*	Tryp E	Forward, 5′ TGCAGACGACCTGACGCTACT 3′	A repetitive nuclear sequence probe pMUTec 6.258	Initial denaturation: 90°C for 7 min 30 cycles of 90°C for 30 s 60°C for 30 s 72°C for 30 s Final extension: 72°C for 7 min	227 bp	(21)
		Tryp E	Reverse, 5′ CTCCTAGAAGCTTCGGTGTCCT 3′				
6	*Mycoplasma* spp.	HBT-F HBT-R	Forward, 5′ ATACGGCCCATATTCCTACG 3′ Reverse, 5′ TGCTCCACCACTTGTTCA 3′	16S rRNA	Initial denaturation: 94°C for 10 min 40 cycles of 95°C for 30 s 60°C for 30 s 72°C for 30 s Final extension: 72°C for 10 min	618 bp (*Candidatus* M. haemominutum), 595 bp (*M. haemofelis*), ~595 bp (any one of *Mycoplasma* spp.)	(22)

### Positive Controls

The DNA isolated from the blood samples (Qiagen DNeasy® blood and tissue kit, Germany) of infected dogs (diagnosed based on microscopical examination of Giemsa's stained blood smears) presented to the Teaching Veterinary Clinical Complex (TVCC), College of Veterinary and Animal Sciences, Pookode, Wayanad were used as positive controls for genus-specific PCRs for *Hepatozoon* spp., *Babesia* spp., and species-specific PCR for *T. evansi*. Polymerase chain reactions specific for *Mycoplasma* spp. and *C. felis* were standardized without any positive controls, as they were rarely reported previously from the state.

### Sequencing and Sequence Analysis

Products of polymerase chain reactions (18S rRNA and 16S rRNA) were purified using NucleoSpin® Gel and PCR Clean-Up Kit (Macherey-Nagel, Germany) as per the manufacturer's protocol. They were sent to the AgriGenome Labs Private Ltd., Cochin, Kerala, for automated nucleotide sequencing by Sanger dideoxy method with both the forward and reverse primers. The resulting sequences were examined for the overlapping peaks suggestive of coinfection using Bioedit software (23) before the comparison of the new sequence of each isolate to other published sequences available in the GenBank using NCBI-BLAST (http://www.ncbi.nlm.nih.gov/BLAST). Unique sequences were deposited in the GenBank database.

### Phylogenetic Analysis

For the phylogenetic analysis, the nucleotide sequences were aligned using ClustalW (24) with the previously published sequences in the GenBank. Aligned sequences were trimmed to the same length (with gaps) from which phylogenetic trees were constructed based on the neighbor-joining (NJ) tree method using the program MEGA X.0 (25) with the suitable models [18S rRNA for *H. felis*: Tamura three-parameter model; 18S rRNA for *Cytauxzoon* spp.: Tamura three-parameter model + gamma distribution (T92 + G = 0.18); 16S rRNA gene of *Mycoplasma* spp.: Kimura two-parameter model]. The reliability of the topologies was tested by bootstrapping with 1,000 replications.

**Table 2:** Sex, breed, and age-wise distribution of hemoparasitic disease and hemotropic mycoplasmosis in domestic cats of Kerala.

**Organism**	**Number of positive cases out of 111 examined**	**Cats**						
		**Sex**		**Breed**		**Age group**		
		**Male**	**Female**	**Persian**	**Nondescript**	**<1 year**	**1–2 years**	**>2 years**
*H. felis*	10 (9%)	7(6.3%)	3 (2.7%)	3(2.7%)	7 (6.3%)	3(2.7%)	4 (3.6%)	3(2.7%)
*Cytauxzoon* spp.	3 (2.7%)	3(2.7%)	–	2(1.8%)	1 (0.9%)	3(2.7%)	–	–
*M. haemofelis*	2 (1.8%)	1(0.9%)	1 (0.9%)	1(0.9%)	1 (0.9%)	–	1 (0.9%)	1(0.9%)
*Candidatus* M. haemominutum	7 (6.3%)	4(3.6%)	3 (2.7%)	2(1.8%)	5 (4.5%)	1(0.9%)	3 (2.7%)	3(2.7%)
*Candidatus* M. turicensis	1 (0.9%)	1(0.9%)	–	–	1 (0.9%)	–	1 (0.9%)	–
Total Feline Mycoplasmosis	10 (9%)	6(5.4%)	4 (3.6%)	3(2.7%)	7 (6.3%)	1(0.9%)	5 (4.5%)	4(3.6%)
Total	23 (20.7%)	16(14.4%)	7 (6.3%)	8(7.2%)	15 (13.5%)	7(6.3%)	9 (8.1%)	7(6.3%)

## Results

### Microscopical Examination

The light microscopy examination of Giemsa's stained peripheral blood smears under oil immersion (100 × ) could not detect any hemoparasites and hemoplasmas in the blood smears of 111 cats examined.

### PCR and Sequence Analysis

None of the samples revealed amplicons specific for the *Babesia* spp. and *T. evansi*. A 358-bp fragment of the 18S rRNA gene of *Hepatozoon*/*Cytauxzoon* species was amplified by PCR using the piroplasm-specific primers from the blood of 13 cats out of the 111 samples examined. Sequencing followed by NCBI-BLAST analysis revealed an identity of 99.4–100% to *H. felis* (JN584475, MK724001) for the 10 sequences and identity of 92.3–92.6% to *C. felis* (GU903911) for the three sequences. These 13 samples were used for confirmation using primers specific for the amplification of the 18S rRNA of *Hepatozoon* spp. and *C. felis*. Ten samples identified as *H. felis* with piroplasmid primers were further confirmed for monoinfection using primers specific for *Hepatozoon* spp. Three samples detected positive, as *Cytauxzoon* spp. did not amplify the desired amplicon when using primers specific for *Hepatozoon* spp., revealing monoinfection in these samples, too. None of the samples produced amplicons specific for the 18S rRNA of *C. felis*.

Amplicons (595, 618, and ~595 bp) specific for the 16S rRNA gene of *Mycoplasma* spp. were amplified by the PCR from the blood samples of 10 cats out of the 111 samples examined. Sequencing revealed that two sequences showed an identity of 98.5–100% to *M. haemofelis* (MK632346, KU645929), seven sequences with an identity of 98.8–99.8% to *Candidatus* M. haemominutum (KU645934, KR905451, MK632386, MK632392) and one sequence with an identity of 99.8% to *Candidatus* M. turicensis (KR905459).

Mixed infection due to the presence of both *H. felis* and *Candidatus* M. haemominutum was identified in 4 sample (3.6%) out of 111 DNA samples by PCR. These cats were from Wayanad (one), Ernakulam (one), and Thiruvananthapuram (two) districts of Kerala. Mixed infection due to different protozoans was not detected in any samples tested in the present study. The occurrence of infection due to hemoparasites and hemotropic mycoplasmas (Table 2) was slightly higher in male compared to female cats. The non-descript cats harbored more infectious organisms than Persian cats. Moreover, cats belonging to the age group of 1–2 years showed a higher prevalence.

## Phylogeny

### Hepatozoon felis

**Figure 2:**
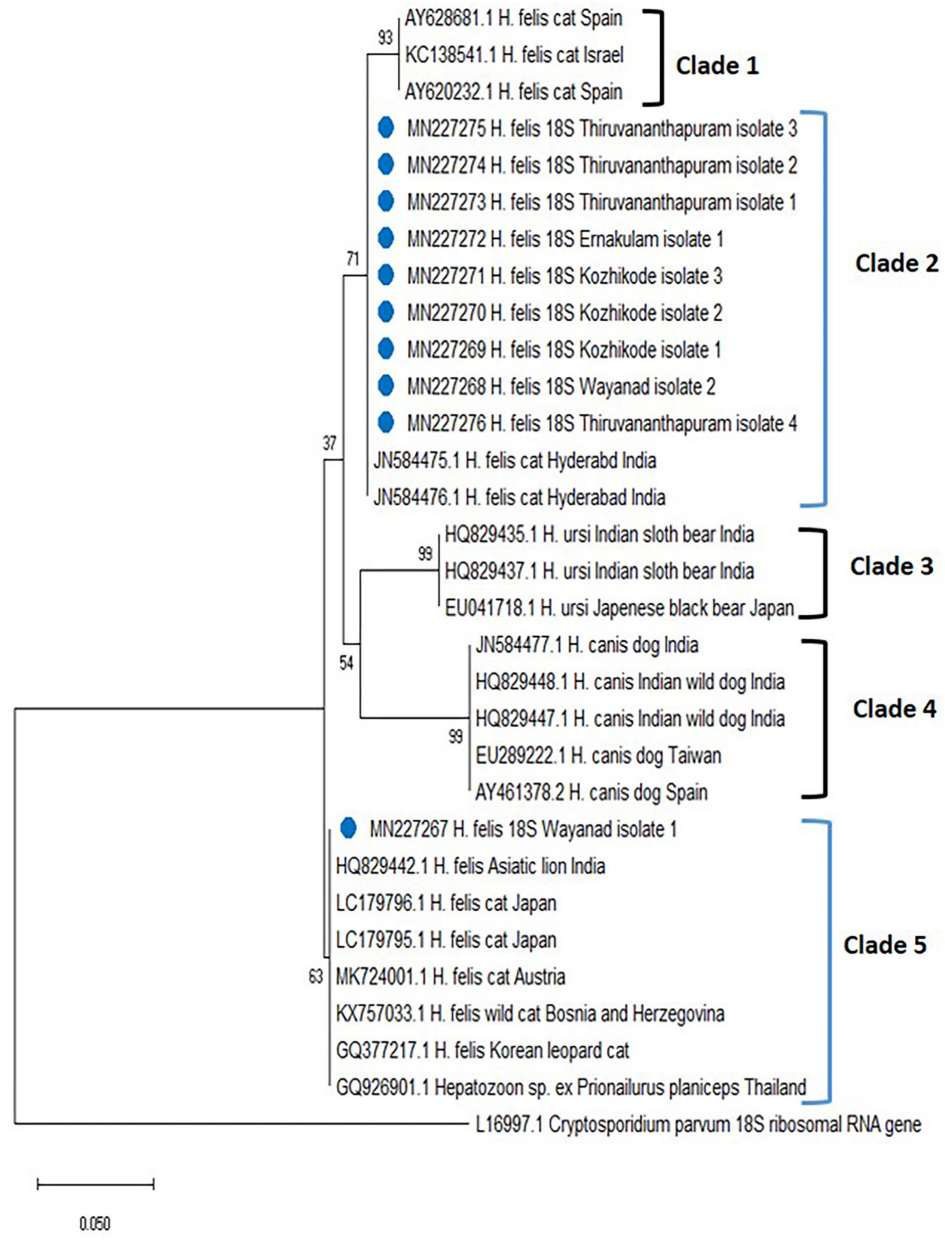
Phylogenetic tree constructed using partial 18S ribosomal RNA (rRNA) gene sequence of *Hepatozoon* spp. amplified using piroplasmid primers. The evolutionary history was inferred by using the neighbor-joining (NJ) tree based on the Tamura three-parameter model (T92). The analysis involved 31 nucleotide sequences comprising of the field isolates from the current study (indicated by the blue circle) and previously published sequences in the GenBank. Evolutionary analyses were conducted in Mega X.

The phylogenetic tree for *Hepatozoon* spp. (Figure 2) based on 18S rRNA sequences revealed five clades (clades 1, 2, 3, 4, and 5). The first clade consisted of *H. felis* sequences from cats of Spain and Israel. Among the 10 isolates of *Hepatozoon* spp., nine (Wayanad isolate 2; Kozhikode isolates 1, 2, and 3; Ernakulam isolate 1; Thiruvananthapuram isolate 1, 2, 3, and 4 with accession numbers MN227268, MN227269, MN227270, MN227271, MN227272, MN227273, MN227274, MN227275, MN227276) clustered with *H. felis* isolates reported from cats of Hyderabad, India in clade 2. The sequences of *Hepatozoon ursi* from the black bear of Japan and the sloth bear of India occupied the third clade. All the *H. canis* sequences from dogs of India, Brazil, Taiwan, and Spain and wild dogs from India were clustered in clade 4. Wayanad isolate 1 of *H. felis* identified in the present study (MN227267) formed a separate clade (clade 5) along with other *H. felis* isolates of domestic cats from Japan and Austria as well as isolates from an Indian lion, a wild cat from Bosnia and Herzegovina, the flat-headed cat from Thailand, and Korean leopard cat.

### *Cytauxzoon* spp.

**Figure 3:**
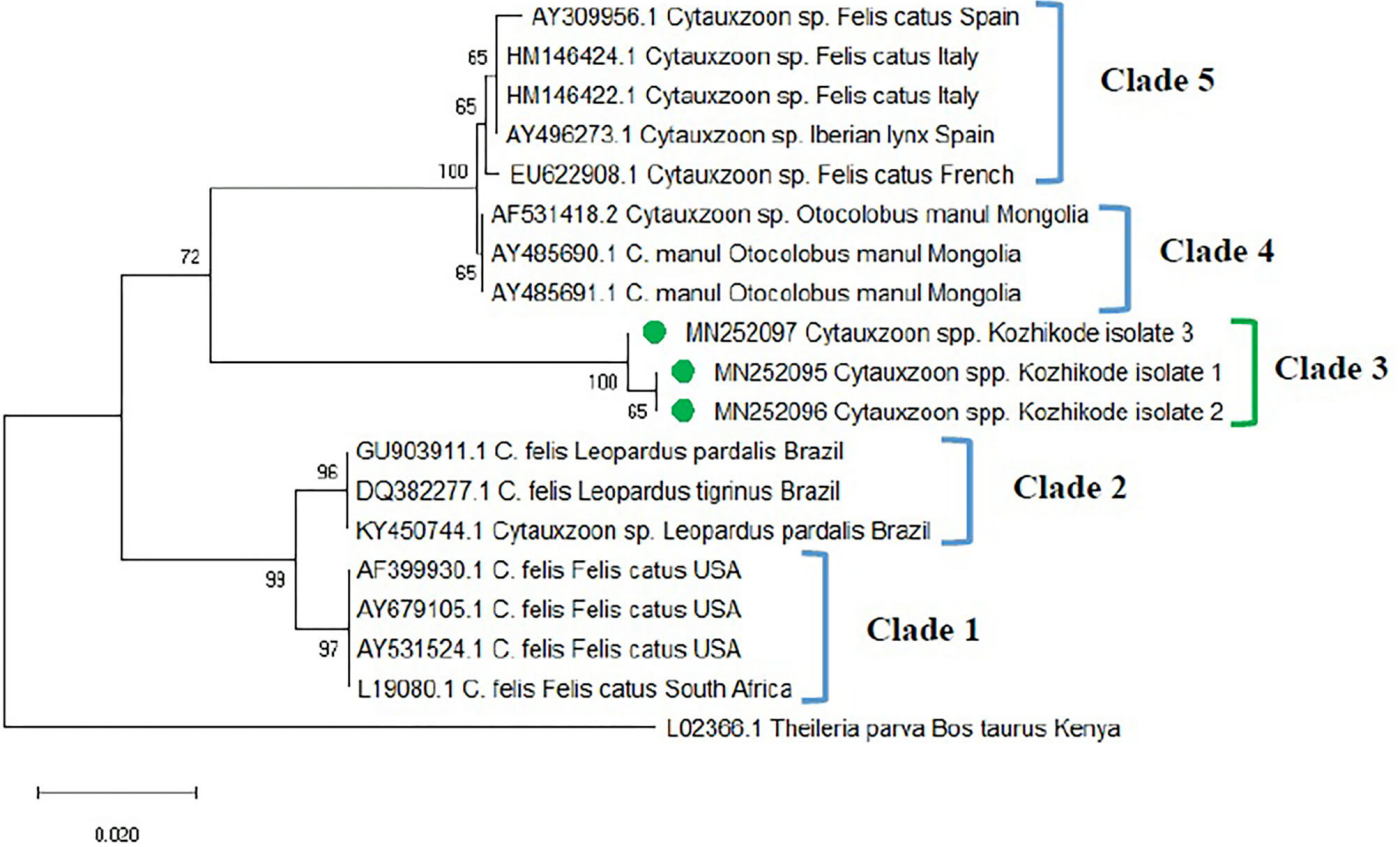
Phylogenetic tree constructed using partial 18S ribosomal RNA (rRNA) gene sequence of *Cytauxzoon* spp. amplified using piroplasmid primers. The evolutionary history was inferred by using the neighbor-joining (NJ) tree based on the Tamura three-parameter model + gamma distribution (T92 + G). A discrete Gamma distribution parameter (+G = 0.18) was used to model evolutionary rate differences among the sites. The analysis involved 19 nucleotide sequences comprising of the field isolates from the current study (indicated by the green circle) and previously published sequences in the GenBank. Evolutionary analyses were conducted in Mega X.

The phylogenetic tree was constructed based on 18S rRNA gene sequences (MN252095, MN252096, MN252097) of *Cytauxzoon* spp. (Figure 3) and revealed five clades. Clade 1 comprised of *C. felis* isolates from domestic cats of the USA and South Africa. Sequences of *C. felis* from Ocelot (*Leopardus pardalis*) and Northern Tiger Cat (*Leopardus tigrinus*) of Brazil, clustered in clade 2. The three isolates of *Cytauxzoon* species from Kerala (Kozhikode isolate 1, 2, and 3) formed a separate clade 3. *Cytauxzoon manul* from Pallas cats of Mongolia was grouped in clade 4. *Cytauxzoon* species of undetermined status (*Cytauxzoon* sp. European strain) from Spain, Italy, and France were clustered into clade 5.

### *Mycoplasma* spp.

**Figure 4:**
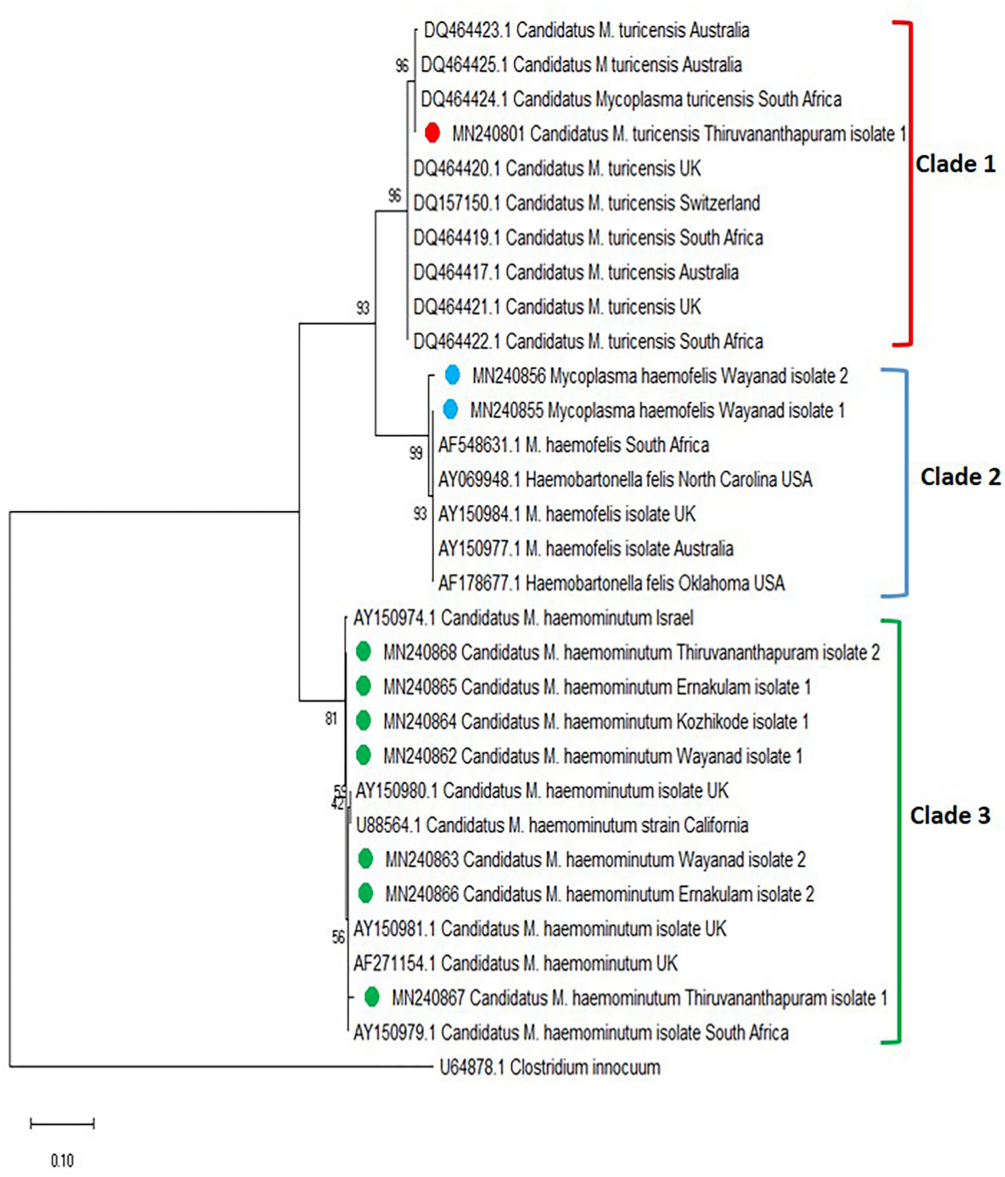
Phylogenetic tree constructed using partial 16S ribosomal RNA (rRNA) gene sequence of *Mycoplasma* spp. amplified using HBT primers. The evolutionary history was inferred by using the neighbor-joining (NJ) tree based on the Kimura two-parameter model. The analysis involved 31 nucleotide sequences comprising of the field isolates from the current study (indicated by the red, blue, and green circle) and previously published sequences in the GenBank. Evolutionary analyses were conducted in Mega X.

The phylogenetic tree was constructed based on the 16S rRNA for *Mycoplasma* spp. [revealed three clades, in which the *Mycoplasma* spp. isolates of Kerala fit into three different clades (clades 1, 2, and 3)] (Figure 4). One sequence of *Candidatus* M. turicensis isolate of Kerala (Thiruvananthapuram isolate 1 with accession number MN240801) clustered in clade 1 along with *Candidatus* M. turicensis isolates from other countries [Australia (DQ464423; DQ464425; DQ464417), South Africa (DQ464424; DQ464419; DQ464422), UK (DQ464420; DQ464421), and Switzerland (DQ157150). Two *M. haemofelis* isolates (MN240855, MN240856) from Kerala (Wayanad isolates 1 and 2) were grouped in clade 2 comprising *M. haemofelis* isolates from other countries (South Africa (AF548631), USA (AY069948; AF178677), UK (AY150984), and Australia (AY150977). Seven isolates of *Candidatus* M. haemominutum from Kerala, *viz*., Wayanad isolate 1 (MN240862), Wayanad isolate 2 (MN240863), Kozhikode isolate 1 (MN240864), Ernakulam isolate 1 (MN240865) and Ernakulam isolate 2 (MN240866), Thiruvananthapuram isolate 1 (MN240867), and Thiruvananthapuram isolate 2 (MN240868) were clustered in clade 3 along with *Candidatus* M. haemominutum isolates from other countries (Israel, UK, USA, and South Africa).

## Discussion

No hemoparasites and hemotropic mycoplasmas could be detected based on microscopy of blood smears stained with Giemsa's stain. *H. felis* gamonts were difficult to be observed under a microscope, as they were less conspicuous, smaller, or low in number (11, 26, 27). The intraerythrocytic piroplasms of *Cytauxzoon* spp. were not detected in healthy cats that were PCR positive (6, 28). It is also believed that the examination of stained smears was not a sensitive diagnostic tool and cannot identify the three different hemoplasma species (29, 30).

In the present study, PCR and subsequent nucleotide sequencing detected 9% prevalence for *H. felis* in the blood samples collected from cats of Kerala. Previously, the presence of *H. canis* (32.3%) in stray cats of Bangkok (31) and *H. felis* (34.8%) and *H. canis* (1.3%) among domestic cats of Israel (17) were reported. Based on 18S rRNA sequences, all the *H. felis* field isolates detected in the present study were grouped into two different clades, clades 2 and 5. The present study also disproved the concept of genetic relatedness of Indian isolates of Hyderabad with isolates from Spain and Israel (15). Thus, there might be two different populations of *H. felis* in Kerala that could infect domestic cats.

The detection of *Cytauxzoon* spp. in three blood samples of cats in the present study forms the first report from India. *C. felis* was endemic solely to North America for many years, where bobcats (*Lynx rufus*) are believed to serve as the main hosts even though reports are available from the USA, Brazil, Spain, France, Italy, and Iraq (32, 33) for its presence in domestic cats. The “parasite” has also been described in felids originating from several Asian countries, including India. However, there are no comprehensive molecular data available, which could confirm the specific identity of these Asian isolates. The nucleotide sequences generated in the current study showed only 92% identity to the closest match in the GenBank database. Furthermore, the Indian sequences formed a separate clade (herein designated as clade 3) that is very distant from that of pathogenic *C. felis* isolates from the USA, Netherlands, and Brazil (6). Further, the primer sets targeting 18 S rRNA gene specific for *C. felis* did not reveal any amplification. The *Cytauxzoon* spp. detected in Kerala were from apparently healthy cats. However, *Cytauxzoon felis* infections (34) are highly fatal except for a few reports from asymptomatic cats (28, 33, 34). In other words, the 18S rDNA sequences confirmed in the cats tested in this study most likely belong to another *Cytauxzoon* species, not *C. felis*.

Hemoplasmosis was previously reported from different parts of the world (7, 29, 30, 35–40). In the present study, *Mycoplasma* spp. was detected in 10 blood samples of cats collected from all four districts of Kerala. Seven cats (6.3%) were infected with *Candidatus* M. haemominutum, two (1.2%) with *M. haemofelis*, and one (0.9%) with *Candidatus* M. turicensis. A previous study conducted in Thrissur, Kerala (16) detected a prevalence of 23% of *Candidatus* M. haemominutum and 1% of *M. haemofelis* by PCR. *Candidatus* M. haematoparvum-like organisms (0.7%) reported previously from the USA (41) were not detected in the present study. In addition, the present study reports for the first time the presence of “*Candidatus* M. turicensis” among the cat population in India.

## Data Availability Statement

The datasets presented in this study can be found in online repositories. The names of the repository/repositories and accession number(s) can be found in the article/Supplementary Material.

## Ethics Statement

The animal study was reviewed and approved by Institutional Animal Ethics Committee, College of Veterinary and Animal Sciences, Pookode. Written informed consent for participation was not obtained from the owners because the samples were collected from cats brought to the veterinary clinics are managed by registered veterinary practitioners. The blood samples were collected by the veterinarians after getting the oral consent from the pet owners for the detection of pathogenic organisms in their pets.

## Author Contributions

LM, AN, CB, BA, PK, RP, and MN collected the samples, conducted the experiments, participated in the data acquisition, and drafted the manuscript. KA conceived the study and supervised the protocols. AV, CD, LJ, and RR helped in the collection of samples, data acquisition, supervision of the experiments, and review of the manuscript. All authors read and approved the manuscript.

## Conflict of Interest

The authors declare that the research was conducted in the absence of any commercial or financial relationships that could be construed as a potential conflict of interest.
